# The Uneven Rate of the Molecular Evolution of Gene Sequences of DNA-Dependent RNA Polymerase I of the Genus *Lamium* L

**DOI:** 10.3390/ijms140611376

**Published:** 2013-05-28

**Authors:** Katarzyna Krawczyk, Jakub Sawicki

**Affiliations:** Department of Botany and Nature Protection, University of Warmia and Mazury in Olsztyn, Plac Lodzki 1, 10-727 Olsztyn, Poland; E-Mail: jakub.sawicki@uwm.edu.pl

**Keywords:** PEP, *rpo*, *Lamium*, molecular evolution, phylogenetic signal, positive-destabilizing selection, adaptative mutations, phylogenetic informativeness

## Abstract

RNA polymerase type I (plastid-encoded polymerase, PEP) is one of the key chloroplast enzymes. However, the *rpo* genes that encode its subunits (*rpoA*, *rpoB*, *rpoC1* and *rpoC2*) are relatively rapidly evolving sequences. The aim of this study was to investigate the rate of the molecular evolution of *rpo* genes and to evaluate them as phylogenetic markers on the example of the genus *Lamium* L. (Lamiaceae). The analyzed genes were shown to differ in the level of variation, rate of intragenic mutations, phylogenetic informativeness, and in the impact of these mutations on the properties of encoded peptides. Destabilizing effects of the positive pressure were observed in all genes examined coding for PEP enzyme. We have demonstrated the relationship between mutations fixed by positive selection and the separation of phylogenetic lines within the genus *Lamium*. The study showed also that the *rpo* genes were reliable phylogenetic markers, useful in the reconstruction of interconnections of species belonging to the same genus. Of the four tested genes, the most promising phylogenetic marker was *rpoA* gene, while the least useful gene appeared to be *rpoC1*.

## 1. Introduction

Plastid-encoded plastid RNA polymerase (PEP), called also RNA polymerase type I, is one of the two DNA-dependent RNA polymerases occurring in chloroplasts of higher plants. In contrast to type II polymerase (nucleus-encoded plastid RNA polymerase, NEP), it is entirely encoded by the chloroplast genome. PEP is involved in tRNA and mRNA synthesis in the chloroplast, thus being one of the key enzymes that maintain the semi-autonomous nature of the plastid [1–3]. Its primary role includes the transcription of photosynthetic genes [4–6]. PEP is constituted by 4 enzymatic subunits: α, β, β′ and β″, which are encoded by the four respective genes from the chloroplast genome, *rpoA*, *rpoB*, *rpoC1* and *rpoC2*.

In spite of performing very important function, the *rpo* genes are relatively fast evolving sequences [7]. For this reason, they have been applied as markers in phylogenetic studies [7–10]. This group of genes has recently received considerable attention also due to their utility in the field of DNA barcoding [11–13]. Previous studies have shown the high relevance of the *rpo* genes in the analysis performed at a high taxonomic level such as division. The analysis of the phylogenetic tree of angiosperms (Magnoliophyta Cronquist) based solely on the *rpo* genes was congruent with the tree built based on complete chloroplast genome [7]. Individual genes of this group have also been applied in the study of evolutionary history at the family level in plants [10,12] and genus level in bacteria [8,9].

The goal of research presented in this paper was to evaluate the *rpo* genes as phylogenetic markers using *Lamium* L. (Lamiaceae) as an exemplar genus. The quality of a phylogenetic marker is determined by both the degree of variation and by the strength and reliability of the phylogenetic signal carried by this DNA region. This signal may be reduced due to the saturation of the tested sequences with substitutions, the nucleotide composition bias or within-site rate variation bias [14]. If these phenomena are pronounced, they can affect the phylogenetic reconstruction, potentially resulting in false though statistically reliable results [14]. In view of the above, we assessed the reliability of the phylogenetic signal carried by the *rpo* genes by analyzing the profile of their phylogenetic informativeness. We also examined the degree of sequences saturation with mutations as well as the variation in the rate of evolution within these genes. The phylogenetic picture of the genus *Lamium*, obtained by analyzing the variability of the *rpo* genes, was compared with findings of previous studies with a larger number of loci [15]. Results achieved enrich the knowledge on the internal phylogenetic relationships of this genus.

The second aim of this work was to complete the phylogeny reconstruction based on nucleotide sequences with information about the evolution at a protein level. For this purpose, we analyzed the impact of substitutions triggering changes of amino acid in the encoded proteins. The effect of substitution depends on the extent of differences in structural and physicochemical properties between the introduced amino acid and the one being replaced. When the selection elicits local but significant changes in protein properties, then this effect is called destabilizing selection [16]. In our research, we have focused our attention on the phenomenon of destabilizing positive selection acting on properties of the corresponding proteins. This is the first study of this type conducted with the use of *rpo* genes.

## 2. Results and Discussion

### 2.1. Sequence Characteristics

**Figure 1:**
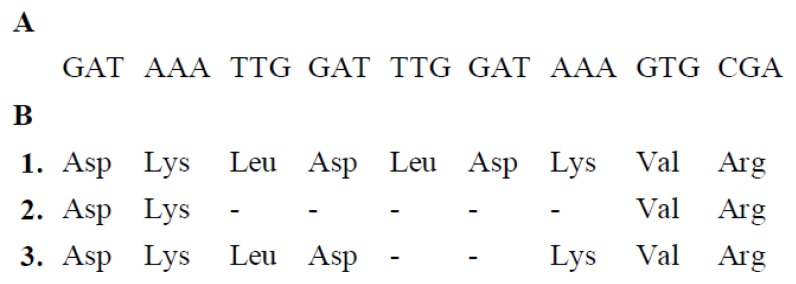
Fragment of the alignment of *rpoA* gene containing indel. (**A**) The nucleotide sequence; sites 688–714; (**B**) The amino acid sequence; sites 230–238. Group 1: *L. album*, *L. tomentosum*; group 2: *L. amplexicaule*, *L. bifidum*, *L. gevorense*, *L. incisum*; group 3: *L. confertum*, *L. coutinhoi*, *L. flexuosum*, *L. galactophyllum*, *L. galeobdolon*, *L. garganicum*, *L.* x *holsaticum*, *L. lycium*, *L. macrodon*, *L. maculatum*, *L. moschatum*, *L. purpureum*, *L. orvala*.

The study was carried out with *rpo* gene fragments ranging from 676 bp (*rpoC2*) to 1269 bp (*rpoB*) in length (Table 1). The variability of *rpoB*, *rpoC1* and *rpoC2* genes was caused exclusively by substitutions, whereas the *rpoA* gene was found to contain one indel (Figure 1). The analyzed sequences of different species could be divided into three groups. The first one consisted of two species: *L. album* and *L. tomentosum*, where the length of the *rpoA* gene fragment was 728 bp. In the second group, consisting of *L. amplexicaule*, *L. bifidum*, *L. gevorense* and *L. incisum*, this region was shorter by 15 bp, which corresponded to five amino acids. The remaining species that constituted the third group, were characterized by the absence of 6 bp when compared to the first group. This indel mutation did not cause changes in the reading frame in any of these groups.

All the *rpo* genes were characterized by a slightly higher content of purines over pyrimidines and by a higher number of AT than GC bonds (Table 1). Among the genes tested, the highest level of variation was detected in the *rpoA* gene. The average rate of its evolution was 1.42 substitutions per nucleotide. The contribution of variable (*V*) and parsimony-informative (*Pi*) sites reached 5.91% and 4.81%, respectively. Slightly less variable was the *rpoC2* gene (*V* = 5.03%, *Pi* = 3.99%) with the substitution rate at 1.15%. The *rpoB* gene was characterized by the lowest proportion of the variable (2.52%) and parsimony-informative sites (1.97%), and by the lowest average rate of substitution introduction (Table 1).

Literature data are lacking that would allow to compare the variation of *rpo* genes of *Lamium* species with that in other genera of vascular plants. We can only refer our results to the few data concerning variability of *rpo* genes within families. Based on a study published by Petersen and Seberg [10], it could be concluded that the degree of variability observed in the *rpoA* gene in the genus *Lamium* (*V* = 5.91%) was higher than in the family Triticaceae (*V* = 4.31%). The variability of *rpoB* and *rpoC1* genes observed in the genus *Lamium* (Table 1) was also higher than in the family Myristicaceae, where the variability of these genes amounted to *V* = 0.82% and *V* = 1.24%, respectively [12]. Although in these families, the variability of genes from the *rpo* group is lower than in the genus analyzed in our study, it is still relatively beneficial when compared with other phylogenetic markers. For example, in the family Myristicaceae, the variability of *rpoC1* was at the similar level as the variability of *matK* gene or *accD* gene [12].

**Figure 2:**
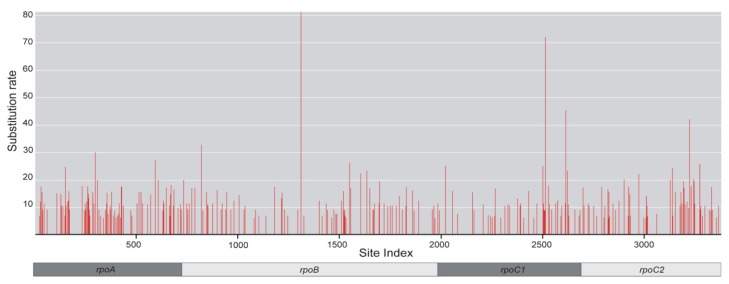
Distribution of substitution rates across *rpo* genes as calculated in HyPhy using the GTR model of evolution. The relative position of sites in analyzed gene fragments is indicated by a bar given below the graph.

Our research showed that the lower average rate of mutations in the *rpoB* and *rpoC1* genes from the *Lamium* genus was correlated with a greater variability of this rate within the genes. Both genes contain vast conserved regions as well as several extremely variable sites (Figure 2).

The *rpoA* and *rpoC2* genes are in this respect much less diverse, which makes their phylogenetic signal more reliable. From the viewpoint of molecular taxonomy, a uniform rate of substitution is preferred within a given segment of the genome [17,18]. The presence of highly variable sites increases the likelihood of reverse mutations or homoplasy, which in turn makes it difficult to correctly reconstruct the phylogenetic relationships [7]. Our observations support findings of investigations carried out at higher taxonomic levels, *i.e.*, in division Magnoliophyta, in which the *rpoA* and *rpoC2* are better phylogenetic markers than *rpoB* and *rpoC1* [7]. The result obtained by Logacheva *et al.* [7] based on the number of correctly resolved nodes of phylogenetic tree, which was adopted as a measure of effectiveness.

### 2.2. Phylogenetic Signal and Informativeness

**Figure 3:**
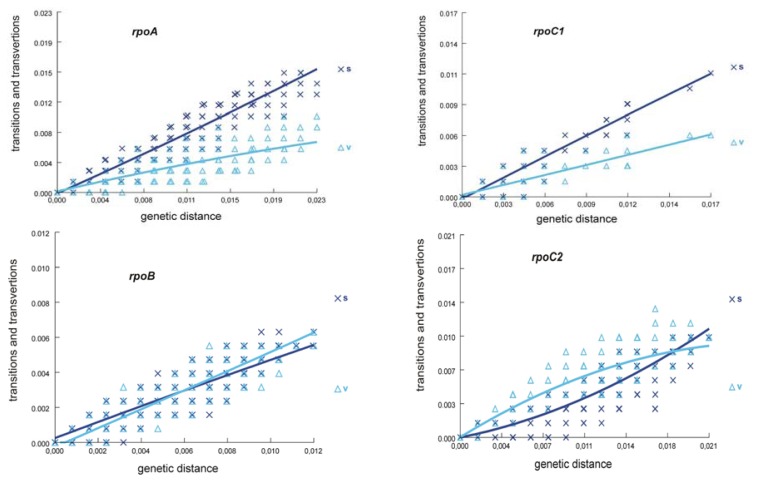
Transitions and transversions *versus* divergence plots (s—transitions, v—transvertions; lines show the general trend). The estimated number of transitions and transversions for each pairwise comparison was plotted against the genetic distance. This pattern can be interpreted as indicating that substitution saturation has not been reached, so that the data can be expected to provide reliable phylogenetic signal.

Individual genome fragments can provide a reliable phylogenetic signal on condition that they have not yet reached the state of substitution saturation. Otherwise, it is difficult to ascertain whether the similarity observed in the pair of sequences is an inherited trait from a common ancestor, or a homoplasy [14,19]. The level of substitution saturation in *rpo* genes was measured by comparing the number of transitions and transversions with the size of the genetic distance for each pair of sequences (Figure 3). The analysis of these genes showed that the amount of substitutions was increasing with the extension of genetic distance. Only the number of transversions within the *rpoC2* gene showed a lower increasing tendency. However, none of the four plots took the form of a plateau, which is typical of the state of saturation with substitutions. Hence, the result achieved speaks for the usefulness of the *rpo* genes in phylogenetic inference in the genus *Lamium*.

**Figure 4:**
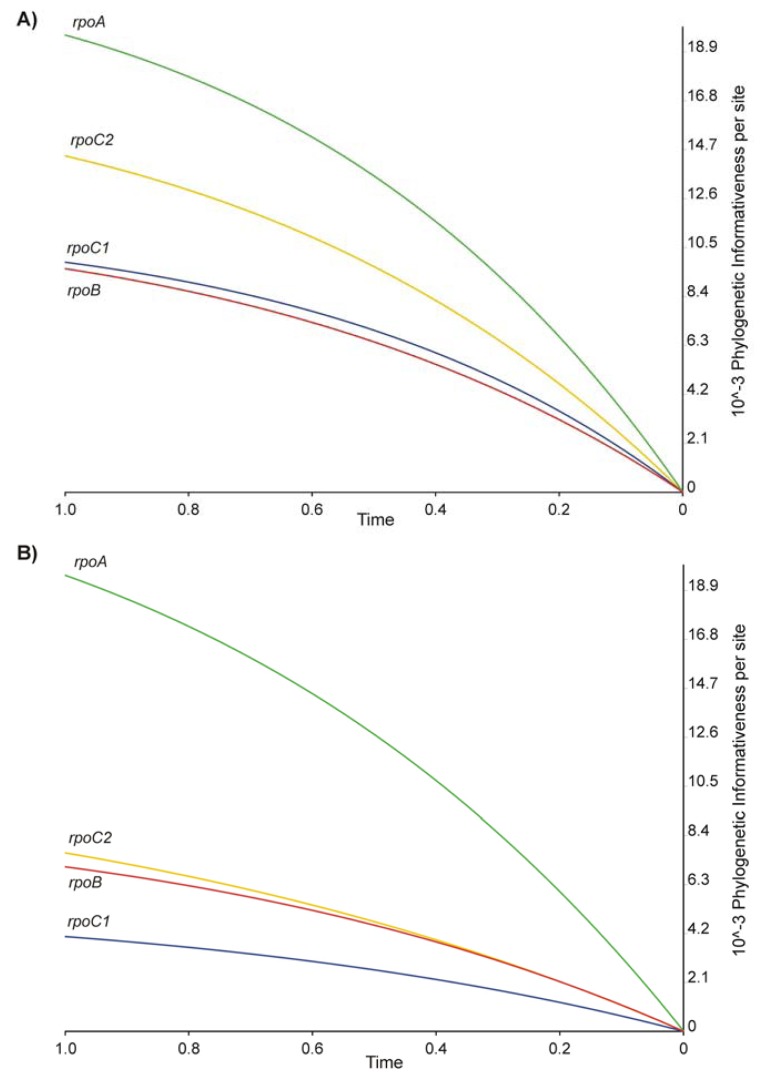
Profile of phylogenetic informativeness for *rpo* genes per site calculated from (**A**) the nucleotide and (**B**) the amino acids sequences (*rpoA*—green, *rpoB*—red, *rpoC1*—blue, *rpoC2*—yellow). The time axis is calibrated in relation to the ultrametrisized ML tree derived from the dataset of four *rpo* genes. An evolutionary time of 1.0 corresponds to the root of the tree and the value of 0 to its tips.

The analysis of the phylogenetic informativeness of individual genes leads to similar conclusions. The PI profiles obtained (Figure 4) demonstrate that the analyzed genes reach the peak of informativeness for events older evolutionarily than the speciation within the genus *Lamium*. This means that the information noise that arose from the accumulation of mutations carrying non-phylogenetic signal was minimized [20,21].

A comparison of the PI profile based on nucleotide sequences with the profile obtained for the amino acid sequences additionally demonstrated the difference in the load of information carried by these two types of data. Caution should, however, be exercised when interpreting graphs shown in Figure 4, as they represent PI values per site. The amino acid sequences are three times shorter than the nucleotide ones, and thus to compare the PI values for these two types of data, results shown on the *Y*-axis of graph in Figure 4B should be divided by three. The results, obtained in this way, showed that the phylogenetic signal carried by all tested genes was significantly stronger at the nucleotide level.

The PI analysis revealed *rpoA* to be the most useful phylogenetic marker for the genus *Lamium* among the genes tested. High informativeness of the nucleotide sequence of the *rpoA* gene (Figure 4A) corresponded with a very strong phylogenetic signal at the amino acid level (Figure 4B). This proved the high convergence of the evolution of a protein sequence with the topology of the phylogenetic tree. Although the *rpoB* gene presented low informativeness at the nucleotide level (Figure 4A), it had a relatively strong phylogenetic signal at the amino acid level (Figure 4B). It could be because the analysis based on the amino acid sequence was less obscured by the information noise compared to the analysis incorporating nucleotide sequences. Therefore, the result of the former analysis was highly reliable even though the amount of information was lower than in the case of nucleotides [22].

There is little data available in the literature on the PI levels of phylogenetic markers in plants. There are no publications that would provide information about the values of PI per site in relation to the genus. For this reason, we could not discuss the level of *rpo* genes informativeness with the results pertaining to the informativeness of other markers at the genus level.

### 2.3. Phylogenetic Tree

**Figure 5:**
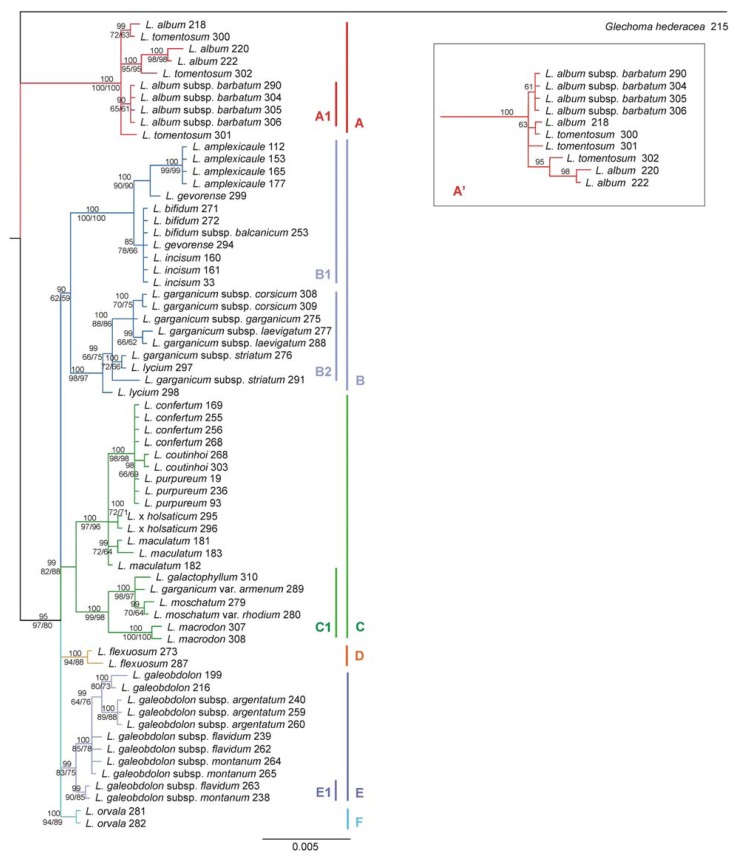
The 50% majority-rule consensus phylogram derived from a Bayesian analysis of *rpo* genes (*rpoA*, *rpoB*, *rpoC1*, *rpoC2*). Credibility values above 0.50 are given in the top line. Bootstrap values of clades supported by maximum parsimony and maximum likelihood analysis are given below (MP value before the slash and ML after the slash). Clades discussed in the text and in Table 2 are marked with a letter. Topography of the clade A obtained with ML method supplied with ML values is shown in a frame as a clade A’.

The phylogenetic tree obtained with the MP method constitutes a consensus of 2258 most parsimonious trees with a length of 294 steps each (*CI* = 0.891473 and *RI* = 0.979651). Its topology is fully consistent with the result of the Bayesian inference. The tree constructed with the ML method was very similar to the remaining two and differed only in topology within the clade A. Cumulative results of the phylogenetic analyses are shown in Figure 5. Phylogenetic analysis based on *rpo* sequence variation does not reflect the full picture of the evolutionary history of the *Lamium* genus. However, it provides valuable information about the phylogenetic relationships between the *Lamium* species examined. The phylogenetic tree of this genus, calculated based on the *rpo* genes, was generally in agreement with results based on the analysis of six, mainly non-coding, chloroplast markers [15].

Our findings demonstrated a large genetic distance between the group comprising *L. album* and *L. tomentosum* (clade A) and the other *Lamium* species. This is indicative of the early separation of this phylogenetic line. Changes in *rpoA*, *rpoB* and *rpoC2* genes in group A were also noticeable at the protein level (Table 2). Group A was additionally interesting because of the relationships of two subspecies, *L. album* and *L. tomentosum*. Among these taxa, only representatives of *L. album* subsp. *barbatum* grouped in one clade, while *L. album* and *L. tomentosum* formed mixed clades. *L. album* ssp. *barbatum* individuality observed at the nucleotide level was confirmed by the presence of an adaptive mutation in a protein encoded by *rpoB* gene. The result obtained with the ML method divided clade A into two smaller clades, thus indicating a greater similarity of *L. album* to *L. tomentosum* than the actual similarity of two subspecies of *L. album* with one another (clade A’). This situation occurred not only in the analysis of the *rpo* genes, but also in that of other chloroplast and nuclear loci [15]. This points to the incomplete lineage sorting of the species discussed. Such a phenomenon occurs when the effective size of the evolving population is large, and the time between successive divisions of phylogenetic lines is too short to fix the mutations that have arisen. It leads to the stochastic sorting of ancestral polymorphisms [23,24].

Our results showed that *L. galeobdolon* (clade E) did not form a separate phylogenetic line in relation to the genus studied and thereby confirmed that the presence of *L. galeobdolon* in the *Lamium* genus was justified. The analysis of *rpo* genes variation supports the hypothesis [15,25,26] that *L. purpureum* is one of the ancestral species of *L. confertum* (clade C). A similar situation applied to *L. bifidum* and *L. incisum* (clade B), where *L. bifidum* is considered the so-called organellar parent of the hybrid species *L. incisum* [15,27–29].

Worthy of special attention in this study was a clear distinction of *L. garganicum* var*. armenum* from other representatives of *L. garganicum*, indicated by a separate, highly supported clade (B2) formed by *L. garganicum* on the phylogram. It was additionally distinguished by three adaptive mutations, which did not appear in other phylogenetic lines (Table 2). The subspecies *L. garganicum* var. *armenum* was located in the clade C, also very reliable statistically. Due to such large differences between these taxa, it was reasonable to exclude *L. garganicum* var. *armenum* from the species *L. garganicum* and adopt its correct synonymous name *L. armenum* Boiss. To our knowledge, the discussed taxon has never been addressed in molecular studies.

### 2.4. Positive Selective Pressure

**Figure 6:**
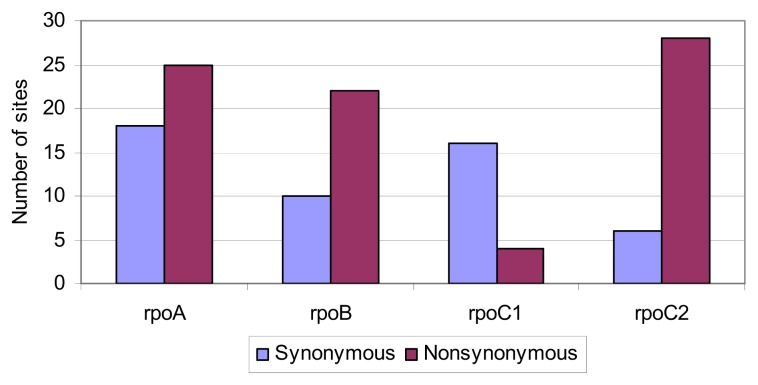
Number of sites containing synonymous and nonsynonymous substitutions (an outgroup not included).

The genes we tested differed in the content of silent mutations and missense mutations. Predominance of variable sites containing synonymous substitutions was reported only for the *rpoC1* gene (Figure 6). Other genes had more non-synonymous sites. Their largest number and the largest percentage in relation to all variable sites was found in the *rpoC2* gene, where out of 34 variable sites, 28 had non-synonymous substitutions.

Part of the observed non-synonymous mutations resulted in significant changes in the structural or physicochemical properties of amino acids. Determining whether the emergence of these changes differed from the assumptions of neutral evolution could indicate which of the properties are affected by selective pressure. The likelihood of positive selective pressure occurrence in particular regions of the *rpo* genes is illustrated with the plots showing the results of the *Z*-test, carried out for the 15-codon-long fragments (Supplementary material 1). Properties of amino acids subjected to positive selective pressure pertaining only to *Lamium* species are summarized in Table 2.

Within the *rpoA* and *rpoC2* genes, the positive pressure was associated with the structural properties of the respective PEP subunits. In the case of *rpoB* and *rpoC1* genes, the pressure focused mainly on the trait described as the equilibrium constant (Table 2). This is a chemical characteristics related to the ionization of a carboxyl group of the amino acid. The formation of a radical mutation usually resulted in a rapid change in one amino acid property. The substitution of cysteine by arginine in the *rpoA* (codon 84) was the only one that resulted in a simultaneous change of two properties, *i.e.*, composition and average number of surrounding residues. When radical mutations were referred to the phylogenetic tree, we observed that they occurred at various organizational levels (Table 2, Figure 5). Some mutations are typical of the multi-species clades, while others–of the species, or even single individuals. One of the mutations observed in the *rpoB* gene (affecting codon 192) occurred simultaneously in three different branches of the cladogram (Table 2).

Our findings showed that the adaptive pressure exerted the greatest influence on the α-subunit of PEP enzyme, encoded by the *rpoA* gene. Within this gene, 25 missense mutations were observed, including 6 resulting from the destabilizing positive pressure. Of these, 4 mutations were associated with well separated clades of the phylogenetic tree. The formation of indel mutations was also significantly correlated with the separation of phylogenetic lines. The group 1 from Figure 1 corresponds to clade A (Figure 5), whilst the group 2 corresponds to clade B1. In the subunit β of PEP (product of *rpoB*), a trait defined as the equilibrium constant (p*K*′) and amino acid 192 were under strong selection pressure (Table 2). As a result, there was a change of isoleucine to methionine, observed as a parallel mutation in clades A1 and F, and in one representative of *L. maculatum*. In the *rpoC2* gene only two mutations could be deemed an outcome of the selective pressure, although this gene contained the highest number of substitutions altering the amino acids. The selective pressure had little effect on the changes occurring in the *rpoC1* gene.

The observed cases of destabilizing positive selection pressure acting on the *rpo* genes indicated that the process of evolution of the PEP enzyme in genus *Lamium* was due not only to the genetic drift. This is evidenced by the evolutionary events that were correlated with the separation of phylogenetic lines of *Lamium* genus. The isolated mutations have been noted in five clades (A1, C, E, E1 and F), and each of these mutations affected one property of the amino acid. In clades A and B2 there were two and three mutations observed, respectively (Table 2). In the clade A, pressure was put on the structural properties (*a*_n_, *P_α_*), while in the clade B2 on two physicochemical traits (pH*_i_* and *pK*′) and one structural property (*N_S_*). Further experimental investigations are needed to estimate the impact of these changes on enzyme functioning. Currently, we can only assume that the natural selection acts to increase the overall efficiency of the enzyme or to optimize its performance in different environments.

## 3. Experimental Section

### 3.1. Plant Material

The study comprised 19 species belonging to the *Lamium* genus, represented by 66 specimens. Different subspecies of *L. album*, *L. bifidum*, *L. garganicum* and *L. galeobdolon*, were also included, which expanded the number of taxa to 28. Nomenclature was adopted from the “World Checklist of Lamiaceae and Verbenaceae” [30]. The exceptions were: *L. garganicum* subsp. *laevigatum* Ces. Pass. & Gibelli, *L. moschatum* var. *rhodium* (Gand.) R.R.Mill., *L. lycium* Boiss., *L. incisum* Willd. and *L. confertum* Fr., which were not considered by these authors as distinct taxonomic units. The names of these taxa were used as described in the herbarium sheets. In the taxonomic approach adopted in our study, *L. galeobdolon* belongs to the genus, however, species transferred by Ryding [31] to the genus *Matsumurella* are excluded. *Glechoma hederacea* L. was included into analysis as an outgroup. Most of the samples were herbarium specimens, and some were collected by the authors during field research (Supplementary material 2).

### 3.2. Experimental Procedures and Sequence Alignment

Leaves were grated in Mini-Beadbeater-1 tissue disruptor and afterwards treated with the Genomic Mini AX Plant SPIN kit (A&A Biotechnology) following the manufacturer’s protocols. The DNA fragments were amplified in a volume of 20 μL containing 20 mM (NH_4_)SO_4_, 50 mM Tris-HCl (pH 9.0 at 25 °C), 1.5 mM MgCl_2_, 1 μL BSA, 200 μM each dATP, dGTP, dCTP, dTTP, 1.0 μM of each primer, one unit of Taq polymerase (Euryx) and 10–20 ng of the DNA template. The reactions were performed under the following conditions: (1) initial denaturation—5 min. at a temperature of 94 °C; (2) denaturation—45 s at 94 °C; (3) annealing—50 s at 55 °C; (4) elongation—1.5 min at 72 °C; (5) final elongation—7 min. at 72 °C. Stages 2–4 were repeated 35 times. Finally, the amplification products were visualized on 2% agarose gel with GelView (Invitrogen™, Carlsbad, CA, USA) staining. Purified PCR products were sequenced in both directions using ABI BigDye 3.1 Terminator Cycle Kit (Applied Biosystems^®^, Foster City, CA, USA) with the same primers and then visualized using an ABI Prism 3130 Automated DNA Sequencer (Applied Biosystems^®^, Foster City, CA, USA). For the amplification and sequencing of *rpoC1* and *rpoC2* we used the primers from Royal Botanical Garden in Kew website [32]. Primers for *rpoA* and *rpoB* genes were designed with the use of chloroplast genome of *Sezamum indicum*, annotated as JN 637766.2 in the GenBank database [33]. The sequences of the applied primers are given in Table 3.

Electropherograms were edited and assembled using Sequencher 4.1.4 (Gene Codes Corporation, Ann Arbor, MI, USA). The assembled sequences were aligned and manually adjusted with BioEdit 7 [34]. Regions of ambiguous alignment and incomplete data (*i.e*., at the beginning and end of sequences) were excluded from the analyses.

### 3.3. Patterns of Substitutions and Phylogenetic Informativeness

The distribution of rates of substitution across sites were estimated using the HyPhy program [35] by assigning a rate class to each site based on the general reversible model. The level of nucleotide substitution saturation was evaluated in DAMBE [36] software by plotting transitions and transversions against pairwise genetic distance. PhyDesign server [19,37] was used to estimate phylogenetic informativeness (PI) of *rpo* genes. PI profiles were plotted with reference to an uncalibrated tree. The tree used to overlay the historic changes in substitution rates was obtained with ML and ultrametrisized using PATHd8 [38]. To obtain relative ages for the clades, the root of the tree was set at an evolutionary time of 1.0 and tips at time of 0. The HyPhy program [35] which is using empirical base frequencies and a time-reversible model of substitution was used to calculate PI of nucleotide data sets. For the aminoacids (AA) data sets the program Rate4Site [39] was used, using a JTT model of evolution and ML inference method. In order to eliminate an influence of gene length on the outcome per site informativeness was calculated.

### 3.4. Phylogenetic Analysis

Bayesian inference (BI), maximum likelihood (ML) and maximum parsimony (MP) methods were applied to infer phylogenetic relationships. Bayesian inference phylogenetic analyses were done using MrBayes v. 3.1.2 [40] with the priors set according to the output of jModelTest 0.1.1 [41]. Optimal models of nucleotide substitution for *rpo* sequences were selected based on Bayesian Information Criterion (BIC) results. The parameters of the likelihood model applied for ITS region were adequate for general time reversible model with a gamma-shaped distribution of rates across sites (*GTR* + *Γ*), (*n_st_* = 6). Bayesian inference was estimated running six incrementally heated chains (MCMC algorithm) for 1,500,000 generations, sampling one out of every 100 generations of random trees. Test run was performed to define the number of generations, which should be excluded from consensus tree calculations. The first 30,000 generations were discarded as “burn-in”. The remaining generations were used to construct the Bayesian consensus tree. The phylogenetic trees generated were graphically adjusted in FigTree v1.3.1 software [42].

ML and MP analyses were conducted using MEGA v.5 [43]. The ML method based on the *GTR* + *Γ* model of nucleotide substitution. In the MP analysis the tree inference was done with Close Neighbor Interchange (CNI) algorithm at the search level of 3 and the number of initial trees equal to 10. The phylogeny was tested both in ML and MP with bootstrap method [44] with the number of bootstrap replications at the level of 1000.

### 3.5. Detection of Positive Selection

The occurrence of a positive selection pressure at the protein level was studied using software TreeSAAP v3.2 [45]. The program measures the selective influence on 31 structural and biochemical amino acid properties across a phylogenetic tree. A gradient of eight categories was used to classify each property change, with lower categories indicating more conservative changes and higher categories denoting more radical changes. Then *Z*-test was performed to determine if certain magnitudes of change deviate from neutral expectations [46]. Positive destabilizing selection on amino-acid property was detected when the frequency of radical changes (6, 7 or 8 category) exceeded the frequency expected by chance, as indicated by positive z-scores. Each of *rpo* genes was tested in TreeSAAP using both the entire data set and a sliding window analysis with 15 codons in width. The results were used to identify the particular amino acid residues that contained positive destabilizing selection for each property.

## 4. Conclusions

Results achieved in our study demonstrated the *rpo* genes to be good phylogenetic markers, useful in the reconstruction of the interconnections of species belonging to the same genus. As coding regions, they contain a highly reliable phylogenetic signal, the value of which can be enhanced by the analysis of changes in the amino acid chain. Another advantage is ease in the alignment of the sequences analyzed. Of the four tested genes, the best performing phylogenetic marker was gene *rpoA*, while the least useful gene appeared to be *rpoC1*. Destabilizing effects of the positive pressure were observed in all genes examined coding for PEP enzyme. We have demonstrated the relation between the mutations fixed by positive selection and the separation of phylogenetic lines within the genus tested.

## Figures and Tables

**Table 1:** Comparison of the analyzed *rpo* gene fragments.

	*rpoA*	*rpoB*	*rpoC1*	*rpoC2*
Alignment length	728	1269	701	676
Sequence length	713–728	1269	701	676
Site rates	1.423	0.773	0.825	1.152
Variable characters (*V*)	43 (5.91%)	32 (2.52%)	20 (2.85%)	34 (5.03%)
Parsimony-informative sites (*Pi*)	35 (4.81%)	25 (1.97%)	18 (2.57%)	27 (3.99%)
Purines %	53.375	53.381	53.458	50.727
Pyrimidynes %	46.625	46.619	46.542	49.273
GC %	37.564	36.311	41.821	43.221
Transition/Transversion bias (*R*)	1.84	1.33	1.73	0.48

**Table 2:** Characteristics of radical amino acid property changes under positive destabilizing selection in *rpo* genes (an outgroup not included).

Gene	Codon	Branch	Amino acid change	Property	Category of change	Statistical error
*rpoA*	79	clade C	C into S	*N*_s_	7	0.010
	128	clade B2	R into I	*N*_s_	6	0.050
	138	clade E	H into Y	*a*_m_	7	0.001
	155	clade B2	R into Q	p*H*_i_	7	0.050
	84	*L. tomentosum* 302	C into R	*C*	7	0.001
	84	*L. tomentosum* 302	C into R	*N*_s_	7	0.010
	110	*L. tomentosum* 301	H into Y	*a*_m_	7	0.001

*rpoB*	90	clade B2	I into L	p*K*′	8	0.010
	160	clade A	S into L	*α*_n_	6	0.010
	192	clade F	I into M	p*K*′	8	0.010
	192	cladeA1	I into M	p*K*′	8	0.010
	192	*L. maculatum* 183	I into M	p*K*′	8	0.010

*rpoC1*	146	clade E1	T into A	*P*_α_	6	0.050
	207	*L. garganicum* subsp. *laevigatum* 277	I into V	p*K*′	8	0.050
	207	*L. garganicum* subsp. *striatum* 291	I into V	p*K*′	8	0.050

*rpoC2*	77	clade A	A into T	*P*_α_	6	0.050
	43	*L. gevorense* 299	A into V	*P*_α_	6	0.050

*N*_s_—average number of surrounding residues; *a*_m_—power to be at the middle of alpha-helix; p*H*_i_—isoelectric point; *C*—composition; p*K*′—equilibrium constant (ionization of COOH); *α_n_*—power to be at the N-terminal; *P*_α_—alpha-helical tendencies.

**Table 3:** Sequences of DNA primers used in the present study.

Region	Forward primer’s sequence (5′-3′)	Reverse primer’s sequence (5′-3′)
*rpoA*	CTACTCGGACACTACAGTGG	GGGAGACAATTCGGATTGATC
*rp*o*B*-1	CCGTCTCTACCCAAGATCCC	GTCGACCAATCCCTTCCTAA
*rpoB*-2	GCGGGGATCCGGTATTTTC	GGCTTTCTAGAGATCCCCAA
*rpoC*1	TATGAAACCAGAATGGATGG	GAAAACATAAGTARRCGWGC
*rpoC*2	CAAAGCAATTTACGCGAAGG	GCAATCACTTGTTCCGATTC

## Abbreviations

PEP: Plastid-encoded plastid RNA polymerase
NEP: Nucleus-encoded plastid RNA polymerase
PI: Phylogenetic informativeness
*Pi*: Parsimony informative
MP: Maximum Parsimony
ML: Maximum likelihood
BI: Bayesian inference
